# The association between dietary fiber intake and gastric cancer: a pooled analysis of 11 case–control studies

**DOI:** 10.1007/s00394-024-03388-w

**Published:** 2024-04-30

**Authors:** Giulia Collatuzzo, Jacqueline Cortez Lainez, Claudio Pelucchi, Eva Negri, Rossella Bonzi, Domenico Palli, Monica Ferraroni, Zuo-Feng Zhang, Guo-Pei Yu, Nuno Lunet, Samantha Morais, Lizbeth López-Carrillo, David Zaridze, Dmitry Maximovitch, Marcela Guevara, Vanessa Santos-Sanchez, Jesus Vioque, Manoli Garcia de la Hera, Mary H. Ward, Reza Malekzadeh, Mohammadreza Pakseresht, Raúl Ulises Hernández-Ramírez, Federica Turati, Charles S. Rabkin, Linda M. Liao, Rashmi Sinha, Malaquias López-Cervantes, Shoichiro Tsugane, Akihisa Hidaka, M. Constanza Camargo, Maria Paula Curado, Nadia Zubair, Dana Kristjansson, Shailja Shah, Carlo La Vecchia, Paolo Boffetta

**Affiliations:** 1 Department of Medical and Surgical Sciences, University of Bologna, Via Massarenti 9, 40138 Bologna, BO Italy; 2 Icahn School of Medicine at Mount Sinai, New York, NY USA; 3 Department of Clinical Sciences and Community Health, University of Milan, Milan, Italy; 4 Fondazione IRCCS Ca’ Granda, Ospedale Maggiore Policlinico, Milan, Italy; 5 Cancer Risk Factors and Life-Style Epidemiology Unit, Institute for Cancer Research, Prevention and Clinical Network, ISPRO, Florence, Italy; 6 Department of Epidemiology, UCLA Fielding School of Public Health and Jonsson Comprehensive Cancer Center, Los Angeles, CA USA; 7 Medical Informatics Center, Peking University, Peking, China; 8 EPIUnit, Instituto de Saúde Pública da Universidade do Porto, Porto, Portugal; 9 Laboratório para a Investigação Integrativa e Translacional em Saúde Populacional (ITR), Porto, Portugal; 10 Departamento de Ciências da Saúde Pública e Forenses e Educação Médica, Faculdade de Medicina da Universidade do Porto, Porto, Portugal; 11 Mexico National Institute of Public Health, Morelos, Mexico; 12 Department of Clinical Epidemiology, N.N. Blokhin National Medical Research Center for Oncology, Moscow, Russia; 13 Consortium for Biomedical Research in Epidemiology and Public Health (CIBERESP), Madrid, Spain; 14 Instituto de Salud Pública y Laboral de Navarra, 31003 Pamplona, Spain; 15 Navarre Institute for Health Research (IdiSNA), 31008 Pamplona, Spain; 16 Centro de Investigacion en Recursos Naturales, Salud y Medio Ambiente, Huelva University, Huelva, Spain; 17 Instituto de Investigación Sanitaria y Biomédica de Alicante, ISABIAL-UMH, 46020 Alicante, Spain; 18 Division of Cancer Epidemiology and Genetics, National Cancer Institute, Rockville, MD USA; 19 Digestive Oncology Research Center, Digestive Disease Research Institute, Tehran University of Medical Sciences, Tehran, Iran; 20 Department of Agricultural, Food and Nutritional Sciences, University of Alberta, Edmonton, AB Canada; 21 Nutritional Epidemiology Group, Centre for Epidemiology and Biostatistics, University of Leeds, Leeds, UK; 22 Department of Biostatistics, Yale School of Public Health, Yale School of Medicine, New Haven, CT USA; 23 Facultad de Medicina, UNAM, 04510 Coyoacán, Mexico; 24 Epidemiology and Prevention Group, Center for Public Health Sciences, National Cancer Center, Chuo City, Tokyo, Japan; 25 National Institute of Health and Nutrition, National Institutes of Biomedical Innovation, Health and Nutrition, Tokyo, Japan; 26 Centro Internacional de Pesquisa, A. C. Camargo Cancer Center, São Paulo, Brazil; 27 Norwegian Institute of Public Health, Oslo, Norway; 28 Division of Gastroenterology, University of California San Diego, San Diego, CA USA; 29 Gastroenterology Section, VA San Diego Healthcare System, San Diego, CA USA; 30 Stony Brook Cancer Center, Stony Brook University, Stony Brook, NY USA; 31 Department of Family, Population and Preventive Medicine, Renaissance School of Medicine, Stony Brook University, Stony Brook, NY USA

**Keywords:** Dietary fiber, Gastric cancer, Gastric neoplasm, Cardia, Non-cardia, Fiber intake

## Abstract

**Purpose:**

Gastric cancer (GC) is among the leading causes of cancer mortality worldwide. The objective of this study was to investigate the association between dietary fiber intake and GC.

**Methods:**

We pooled data from 11 population or hospital-based case–control studies included in the Stomach Cancer Pooling (StoP) Project, for a total of 4865 histologically confirmed cases and 10,626 controls. Intake of dietary fibers and other dietary factors was collected using food frequency questionnaires. We calculated the odds ratios (OR) and 95% confidence intervals (CI) of the association between dietary fiber intake and GC by using a multivariable logistic regression model adjusted for study site, sex, age, caloric intake, smoking, fruit and vegetable intake, and socioeconomic status. We conducted stratified analyses by these factors, as well as GC anatomical site and histological type.

**Results:**

The OR of GC for an increase of one quartile of fiber intake was 0.91 (95% CI: 0.85, 0.97), that for the highest compared to the lowest quartile of dietary fiber intake was 0.72 (95% CI: 0.59, 0.88). Results were similar irrespective of anatomical site and histological type.

**Conclusion:**

Our analysis supports the hypothesis that dietary fiber intake may exert a protective effect on GC.

**Supplementary Information:**

The online version contains supplementary material available at 10.1007/s00394-024-03388-w.

## Introduction

Gastric cancer (GC) is among the most commonly diagnosed cancers, and it is the 4th leading cause of cancer mortality globally [1]. In addition, GC has the highest incidence rates in East Asia, South America, and East Europe [2]. The risk factors for GC include *Helicobacter pylori* (*Hp*) infection, cigarette smoking, alcohol consumption and high salt intake [2]. High consumption of red meat is also a risk factor [2, 3]. The stomach is divided into anatomical parts, namely cardia and non-cardia. There are two main histological types of GC, namely intestinal and diffuse, with different epidemiological features [4].

Certain dietary factors, including fibers, have been be associated with a reduced risk of GC [5, 6]. Dietary fibers are known to regulate the speed, bulk and consistency of stools and contribute to the equilibrium of the microbiota [7]. Fibers are fermented into short-chain fatty acids such as butyrate, which can in turn be implicated in gene regulation, by inhibiting cell proliferation and therefore exerting a tumor suppressive effect [8]. Dietary fiber can therefore have beneficial effects on the risk of some chronic diseases and cancers, including colorectal cancer [9, 10] According to a meta-analysis by Zhang et al., fibers are associated with a 40% decreased risk of GC [11]. Anyway, this study is relatively old and the authors reported significant heterogeneity among the included studies [11]. Subsequent studies confirmed the protective role of fiber on GC [12–15]. However, other authors did not find any association [16].

Given the potential importance of dietary fiber on health, our aim was to investigate the association between dietary fiber intake and GC risk, including its association with different anatomical subsites and histological types of GC in a large pooled dataset. Moreover, given the potential confounding underlying this association, the analyses we conducted were aimed at disentangling the effect of fibers from that of fruit and vegetable intake, which are among the main dietary source of fibers, in order to assess whether this important dietary component is specifically associated to GC.

## Methods

Information and data for this study were derived from the Stomach Cancer Pooling (StoP) Project, an international consortium of 34 case–control and nested-case control studies on GC which collected epidemiological data to investigate associated factors [17]. Potentially relevant studies are identified through literature searches and principal investigators are invited to join the consortium and share original data on sociodemographic, clinical and lifestyle factors which are known or suspected risk factors for GC. For the purpose of data harmonization, the data were split into several sections (sociodemographic characteristics, tobacco smoking, Hp infection, etc.) and a codebook was created for each topic. The data were then standardized for the variables included in each analysis of the consortium. Completeness and consistency of the variables were centrally checked. Implausible and inconsistent values as well as outliers were checked in collaboration with original investigators. The StoP Project received ethical approval from the University of Milan Review Board (reference 19/15 on 01/04/2015).

A total of 14 studies included information on dietary fiber intake. We excluded from the analysis one study whose mean dietary fiber intake was low, since all subjects were classified in the lowest quartile of the pooled distribution [18], and two additional studies which lacked data on key confounders [19, 20] (see below). We therefore included 11 case–control studies in the pooled analysis, which were conducted in Italy (2 studies), Russia, Iran, China, Portugal, Spain (2 studies), Mexico (2 studies) and the United States [21–31]. Three of the studies were hospital-based, while the remaining studies were population-based. Other selected characteristics of these 11 studies included are reported in Supplementary Table 1.

Cases were defined based on histologically confirmed GC. When available, we considered as outcome specific anatomical subsite (cardia and non-cardia) and histological type (intestinal and diffuse) of GC.

The primary exposure was dietary fiber intake, determined by food frequency questionnaires (FFQ), which were used along with country-specific food composition tables to calculate intake of nutrients. Total dietary fiber intake was measured in grams per day, and categorized into quartiles based on the overall fiber consumption of the pooled population. Subjects with extreme values of caloric intake (< 500 and > 5000 kcal/day) and subjects with less than 1 g of fiber/day intake were excluded from the analysis, leaving 4865 cases and 10,626 controls.

### Statistical analysis

A multivariable logistic regression was conducted to calculate the odd ratio (OR) and the corresponding 95% confidence interval (CI) of the association between fiber intake and GC. Variables for adjustment were selected based on (i) data availability in the included studies, (ii) scientific rationale, and (iii) change in the OR for fiber by at least 10%. In preliminary analyses, we assessed the potential confounding effect of sex, age, tobacco smoking, alcohol consumption, total energy intake, fruit and vegetable intake, salt intake, meat intake and socioeconomic status. Hp infection was not included in the analysis due to the large number of missing values. We excluded alcohol drinking and salt intake, since in preliminary analyses they did not appear to act as confounders, while they were missing from some of the studies selected for the analysis. Meat intake was available for a subset of studies, and was not retained in the main analysis, Total energy intake and fruit and vegetable intake, on the other hand, did appear to be confounders of the association between fiber intake and GC, and were retained in the final regression model, even if they were missing from two of the studies [19, 20], which were therefore excluded from the pooled analysis.

The final regression model included study country, sex, age (< 55, 56–65, 66–75, 76 +), tobacco smoking status (never, former, current smoker), total calorie intake (quartiles: 500–1597 kcal/day, 1598–2045 kcal/day, 2046–2565 kcal/day, ≥ 2566–5000 kcal/day), fruit and vegetable intake (based on tertiles of the distribution among controls), and socioeconomic status (based on study-specific categories). Subjects with missing data were excluded from the analysis. The primary analysis was conducted on the pooled dataset. To assess the robustness of the results, we repeated the analysis using a two-stage approach, in which the study-specific ORs, obtained according to the model specified above, were combined using a random-effects meta-analysis [32].

We also conducted stratified analyses by each of the variables included in the final model, as well as anatomical subsite (cardia and non-cardia), and histological type (intestinal and diffuse) of GC. To verify the validity of our results, we conducted sensitivity analyses based on study-specific quartiles of dietary fiber intake, and repeating the primary analysis after excluding one potential confounder at a time from the regression model. Moreover, we repeated the main analysis without adjusting for fruit and vegetable intake, in order to identify potential influence of this variable on the association between dietary fiber intake and GC. This, in other words, was used as a surrogate of fiber source, which was not available, to be able to disentangle the effect of dietary fiber intake from that of fruit and vegetable.

For completion, the main analysis was repeated with and without adjusting each time for Hp (negative vs positive), alcohol (never, < = 12 gr/day, 12–47 gr/day, > 47 gr/day), salt (low, medium and high intake), meat (< = 55, 55–101, 101–500 g/day), and vegetables and legumes (low, intermediate, high intake based on based on tertiles of the distribution among controls). A variable for fruit only was also categorized in study-specific tertiles (based on tertiles of the distribution among controls). In the model accounting for vegetables and legumes intake, fruit was considered as a separate term categorized in tertiles of intake.

All statistical analyses were performed on STATA, version 16.1 (Stata Corp., College Station, TX, US). [33].

The pooled analysis within the StoP Consortium was approved by the Ethics Committee of the University of Milan.

## Results

The analysis included 15,491 individuals recruited in 11 case–control studies, of whom 4865 (31.4%) were cases and 10,626 (68.6%) controls. Selected characteristics of these subjects are reported in Table 1. Compared to controls, cases had higher caloric intakes, lower intakes of fruits and vegetables, and had a higher proportion of smokers, and a lower socioeconomic status. Details on the distribution of fiber intake in each study are reported in Supplementary Table 2: the mean intake ranged from 5.8 g/day [24] to 39.4 g/day [23].

**Table 1:** Selected characteristics of cases and controls included in the analysis

Characteristic*	Cases (N = 4865)	Controls (N = 10,626)	*p*-value
Sex†			–
Male	3020 (62)	5752 (54)	
Female	1845 (38)	4874 (46)	
Age†			
< 55	1173 (24)	3113 (29)	–
56–65	1306 (27)	2922 (28)	
66–75	1783 (37)	3285 (31)	
> 76	603 (12)	1392 (12)	
Fiber intake (g/day)			< 0.001
1.0–14.1	1398 (30)	2229 (22)	
14.2–20.7	883 (19)	2550 (25)	
20.8–28.9	879 (19)	2647 (26)	
29.0 +	1514 (32)	2668 (26)	
Total energy (kcal/day)			< 0.001
500–1597	1077 (23)	2558 (25)	
1598–2045	857 (18)	2503 (25)	
2046–2565	1107 (24)	2509 (25)	
2566 +	1599 (34)	2465 (25)	
Fruit and vegetable intake			< 0.001
Lower tertile	1663 (36)	2943 (30)	
Middle tertile	1514 (32)	3319 (34)	
Upper tertile	1498 (32)	3613 (37)	
Tobacco smoking			< 0.001
Never smoker	2265 (48)	5092 (49)	<
Former smoker	1191 (25)	2782 (27)	
Current smoker	1311 (27)	2564 (25)	
Socioeconomic status			< 0.001
Low	2858 (60)	4928 (47)	
Medium	1489 (31)	3532 (34)	
High	436 (9)	2031 (19)	

*Sums might not add to the total because of missing values

^†^*p* values for sex and age are not reported as cases and controls were matched for these variables

Results of the main analysis are shown in Table 2. The OR of GC were 0.79 (95% CI 0.68–0.91), 0.72 (95% CI 0.60–0.85) and 0.72 (95% CI 0.59–0.88) for the second, third and fourth quartile of fiber intake compared to the first quartile. The OR per one quartile increase was 0.91 (95% CI 0.85, 0.97), and was 0.96 (95% CI 0.91–1.01) per 10 g/day increase of fiber intake. The results of the analysis of individual studies and of their meta-analysis are reported in Supplementary Table 3: although there was some heterogeneity in study-specific results (p-value of test for heterogeneity 0.005), the results of the meta-analysis were congruent with those of the pooled analysis reported in Table 2.

**Table 2:** Odds ratio of gastric cancer for fiber intake

Fiber intake (g/day)	N ca/co	OR^a^	95% CI
1.0–14.1	1398/2227	1.00	Ref.
14.2–20.7	883/2550	0.79	0.68–0.91
20.8–28.9	879/2647	0.72	0.60–0.85
29.0 +	1514/2668	0.72	0.59–0.88
Per quartile increase		0.91	0.85–0.97
Per 10 g/day increase		0.96	0.91–1.01

*OR* odds ratio, adjusted for study, sex, age, tobacco smoking, fruit and vegetable intake, total energy intake, socioeconomic status; *CI* confidence interval; *Ref*. reference category; *N ca/co*, number of cases and controls

Results of the analysis stratified for potential effect modifiers are shown in Table 3. There was no modification of the association between fiber intake and OR of GC according to sex, age, total energy intake, tobacco smoking or socioeconomic status. On the other hand, there was a suggestion of a modification of the effect of fiber intake according to fruit and vegetable intake, with an association between fiber intake and GC risk present only among subjects in the upper tertile of fruit and vegetable intake, although the test for heterogeneity was not statistically significant after accounting for multiple comparisons.

**Table 3:** Odds ratio of gastric cancer for fiber intake (per quartile increase), stratified by selected characteristics

Fiber intake (per quartile increase)	OR	95% CI	*p* heter
Sex			0.75
Men	0.91	0.84–0.99	
Women	0.93	0.84–1.04	
Age			0.98
< 55	0.91	0.80–1.03	
56–65	0.91	0.80–1.03	
66–75	0.92	0.82–1.04	
76 +	0.88	0.73–1.06	
Total energy (kcal/day)			0.73
500–1597	0.84	0.73–0.96	
1598–2045	0.88	0.78–0.99	
2046–2565	0.92	0.82–1.04	
2566 +	0.92	0.82–1.04	
Fruit and vegetable intake			0.02
Lower tertile	1.00	0.89–1.13	
Middle tertile	1.04	0.92–1.18	
Upper tertile	0.83	0.73–0.93	
Tobacco smoking			0.30
Never smoker	0.90	0.82–0.99	
Former smoker	0.98	0.86–1.11	
Current smoker	0.85	0.75–0.97	
Socioeconomic status			0.82
Low	0.91	0.83–1.00	
Medium	0.87	0.78–0.97	
High	0.91	0.74–1.12	

*OR* odds ratio, adjusted for study, sex, age, tobacco smoking, fruit and vegetable intake, total energy intake, socioeconomic status, as appropriate; *CI* confidence interval; *Ref*. reference category; *p het p*-value of test for heterogeneity between strata

Results of the analyses by GC anatomical site and histology are reported in Table 4, although the small number of GC cases precluded robust analyses. there was no heterogeneity according to anatomical site. The association between fiber intake and diffuse GC appeared to be stronger than that with intestinal GC; there was no statistical evidence of heterogeneity.

**Table 4:** Odds ratio of gastric cancer for fiber intake, by anatomical site and histology*

Fiber intake (g/day)	Anatomical site				Histology			
	Cardia (N = 440)		Non-cardia (N = 2735)		Intestinal (N = 1601)		Diffuse (N = 973)	
	OR	95% CI	OR	95% CI	OR	95% CI	OR	95% CI
1.0–14.1	1.00	Ref.	1.00	Ref.	1.00	Ref.	1.00	Ref.
14.2–20.7	0.63	0.43–0.91	0.72	0.60–0.87	0.71	0.57–0.89	0.64	0.50–0.84
20.8–28.9	0.56	0.36–0.85	0.64	0.52–0.79	0.70	0.54–0.91	0.55	0.40–0.76
29.0 +	0.66	0.40–1.08	0.69	0.57–0.83	0.82	0.61–1.12	0.59	0.41–0.86
Per quartile increase	0.90	0.77–1.06	0.91	0.84–0.98	0.97	0.88–1.07	0.87	0.78–0.98
*p*-het	0.90				0.16			

*N* number of cases; *OR* odds ratio, adjusted study, sex, age, tobacco smoking, fruit and vegetable intake, total energy intake, socioeconomic status; *CI* confidence interval; *Ref*. reference category; *p-het*, *p*-value of test of heterogeneity between strata

*Information not available for studies [17, 18, 20]. Missing information on anatomical site for 589 cases, on histology for 1190 cases

The results of the sensitivity analysis based on study-specific quartiles of fiber intake suggested an inverse association with GC risk, although the decrease in the OR was less pronounced than in the primary analysis (Table 5). The primary analysis was repeated using these study-specific quartiles and excluding one potential confounder at a time from the regression model (Supplementary Table 4). The factors which appeared to exert a confounding effect were fruit and vegetable intake and total energy intake. In particular, the effect of fiber intake was stronger in the analysis based on models that did not include a term for fruit and vegetable intake.

**Table 5:** Odds ratio of gastric cancer for study-specific quartiles of fiber intake

Fiber intake	OR	95% CI
Quartile 1	1.00	Ref.
Quartile 2	0.92	0.82–1.03
Quartile 3	0.92	0.81–1.03
Quartile 4	0.87	0.75–1.01
Per quartile increase	0.96	0.92–1.01

*OR* odds ratio adjusted study, sex, age, tobacco smoking, fruit and vegetable intake, total energy intake, socioeconomic status; *CI* confidence interval; *Ref*. reference category

Data on Hp were scarce and impaired by reverse causation, leading to a negative relationship between Hp and GC, however the effect of fiber on GC did not change in the models adjusted and non-adjusted for this risk factor (not shown in detail). The effect of fiber was maintained also in models accounting for vegetables and legumes intake, alcohol intake and salt intake, while meat intake hid its protective role on GC (Supplementary Table 5).

## Discussion

Our pooled analysis included studies conducted in eight countries from three continents. Although there was some heterogeneity between the studies, the majority of the results indicated a reduction in GC risk with increasing dietary fiber intake. Given the extensive effort within the StoP Consortium to harmonize individual data across studies, our analysis provides stronger evidence than meta-analytic approaches based on grouped data [34].

Based on the results of this study, dietary fiber intake is associated with a decreased risk of gastric cancer. This was also found to be true across anatomical subsites and, although based on small numbers, for the diffuse histologic type. In the pooled analysis, a reduced OR was observed in the highest quartile of fiber intake, and an inverse dose–response relationship was identified.

Our results are consistent with the conclusions of the Third Expert Report by the World Cancer Research/American Institute for Cancer Research [35]. That report notes that high dietary fiber intake contributes to the prevention of cancer, including that of the stomach. The results are also consistent with a meta-analysis published in 2013, whose authors found an inverse association between fiber intake and risk of GC [11], and cohort studies [12, 13, 15]. We could assess the independent effect of dietary fiber intake from that of fruit and vegetables on GC, offering results both adjusted and unadjusted for these terms. Also, this is one of the few studies to provide data on different types of GC.

The recommended intake of fiber is 25–35 g/day [31]. In the US, a mean intake of 16 g per day was reported in the 2009–2010 National Health and Nutrition Examination Survey [36]. Lower intakes have been reported in other populations, e.g., 9.7 g/day in China in 2015 [37]. In our study, the mean intake of fiber was 22.7 g/day, with small differences between cases and controls.

Our data showed there is an inverse dose–response relationship between dietary fiber intake and GC, after adjusting for study, sex, age, total calorie intake, fruit and vegetable intake, tobacco smoking and socioeconomic status. It can be argued that fruit and vegetable intake should not be considered a potential confounder, since these foods represent a major source of fibers. In this respect, the risk estimates for dietary fiber intake, after adjustment for fruit and vegetable intake, mainly reflect the effect of dietary fiber intake from other sources, such as whole grains. The observation that the association with fibers is less strong after adjustment for fruit and vegetables is due to the fact that these fruit and vegetables are major sources of fibers. In this respect, it is of interest to notice that a residual effect of dietary fiber intake is still apparent among subjects in the highest tertile of fruit and vegetable intake when accounting for vegetables and fruit intake. We further tested this by adjusting for legumes intake, obtaining results similar to the main analysis. Overall, one can conclude that dietary fiber intake appears to exert a protective effect in this analysis, irrespective of their source. When study-specific quartiles were used, there was an inverse dose–response relationship between fiber and GC similar to that detected in the analysis based on pooled categories. No effect modification by sex, age, tobacco smoking, total energy intake or socioeconomic status was identified.

Furthermore, when we adjusted for other possible dietary confounders such as alcohol and salt, we still observed a protective effect of fiber. The association was weakened onlywhen accounting for meat. These elements add to the internal consistency of the results.

Fibers have been studied in association to gastrointestinal cancer, with many studies reporting their potential beneficial effects. The findings on the associations of fibers and GC have been inconsistent. In a study based on a multicenter European cohort, fruit but not vegetable intake was inversely associated with GC risk, and there was no association between total fiber intake and GC [12]. A prospective study conducted in Japan also reported a null association between fiber intake and GC [13]. Conversely, large case–control study from Sweden found a strong dose-dependent protective effect between fiber intake and cardia GC. In particular, this favorable effect was mainly related to cereal fiber intake, while fibers derived from fruits and vegetables were not associated with GC [38]. A further large study from Canada reported a weak inverse association, with small differences by anatomical subsite [39].

An umbrella review from 2018 found inverse relationship between GC and dietary fiber with an OR of 0.57 (95% CI 0.49–0.67) for the comparison of the highest versus the lowest category of intake, based on 26 cohort and case-controls studies [40]. Additionally, this review suggested that the favorable role of fiber on GC may be related to their anti-inflammatory properties, and their favorable interaction with gut microbiota. Part of the effect of fiber may be driven by their micronutrient composition, such as vitamins, minerals and phytoestrogens [40]. Capuano explained the role of fibers derives from the digestion process rather than their intrinsic nutritional value [41]. Physicochemical properties of fiber, like particle size, solubility, hydration properties, and viscosity may determine their effect on human health. These characteristics may change based on additional factors, such as concomitant intake with other foods, and modality of administration of fibers (i.e. through beverage) making the prediction of their effect rather complex.

This study has several strengths. We pooled individual-level data from a number of international studies, providing stable summary risk estimates. We explored detailed aspects of the association between dietary fiber intake and GC risk, including the effect on anatomical subsites and histological types, the interaction with other risk factors of GC, and their potential residual confounding. Notably, we could test several confounders, and presented solid results which were corroborated in secondary and sensitivity analyses.

This study also has limitations. First, selection bias is a potential limitation of community-based case–control studies, in particular those with hospital-based design. Also, recall bias for dietary and other factors, which are known to be linked to case–control studies, mighthave also affected the results. This should in particular be accounted when comparing our results with cohort studies [12, 13, 15]. The food composition tables used to convert the data from food frequency questionnaires into an estimate of grams per day were based on country averages, which could also lead to misclassification. However, the fact that our results are consistent with those of other large studies and meta-analyses [35] suggests that exposure misclassification did not play a major role. In addition, we could not distinguish between sources of dietary fiber that may have led to these effects, nor separating soluble and insoluble fibers. This analysis had limited information on *Hp* status, which is an important risk factor for GC, that could contribute to residual confounding. The majority of the studies in the consortium are of case–control design, and information on Hp status are collected after GC diagnosis in the cases. This leads to an unlikely low rate of Hp positivity among the cases due to atrophy of stomach mucosa, leading to disappearance of the infection (reverse causality) [42]. Future studies should include different areas of the world and collect data on specific foods, sources, and types of fiber. Results by anatomical subsite and histological type should be interpreted with caution, given the relatively limited sample size.

In conclusion, this study supports the hypothesis that dietary fiber intake has a favorable effect on GC. Further analyses should be conducted to clarify the causal nature of this association.

### Supplementary Information

Below is the link to the electronic supplementary material.

Supplementary file1 (DOCX 17 KB)

## Data Availability

Deidentified data will be made available upon reasonable request.
