# Supplementary material for: The association between dietary fiber intake and gastric cancer: a pooled analysis of 11 case–control studies
Source: Eur J Nutr. 2024 Apr 30;63(5):1857–65. doi: 10.1007/s00394-024-03388-w (PMC11329417; doi:10.1007/s00394-024-03388-w)
Supplement: Supplementary file 1 — Supplementary file1 (DOCX 17 KB) [file 394_2024_3388_MOESM1_ESM.docx]

Supplementary Table 1. Selected characteristics of studies included in the analysis

| Study | Country | Study Period | Cases (N) | Controls (N) | Study Design |
| --- | --- | --- | --- | --- | --- |
| Buiatti et al., 1989 [16] | Italy | 1985-1987 | 1016 | 1159 | Population-based |
| Ward et al., 1997 [17] | USA | 1988-1993 | 170 | 502 | Population-based |
| Lopez Carillo et al., 1999 [18] | Mexico | 1989-1990 | 220 | 752 | Population-based |
| Zaridze et al., 2000 [19] | Russia | 1996-1997 | 450 | 611 | Hospital-based |
| Setiawan et al., 2005 [20] | China | 1991-1993 | 711 | 711 | Population-based |
| Lunet et al., 2007 [21] | Portugal | 1999-2006 | 692 | 1667 | Population-based |
| Pakseresht et al., 2007 [22] | Iran | 2005-2007 | 286 | 304 | Population-based |
| Lucenterforte et al., 2008 [23] | Italy | 1997-2007 | 230 | 547 | Hospital-based |
| Hernandez Ramirez et al., 2009 [24] | Mexico | 2004-2005 | 248 | 478 | Population-based |
| Santibanez et al., 2012 [25] | Spain | 1995-1999 | 401 | 455 | Hospital-based |
| Castano Vinyals et al., 2015 [26] | Spain | 2008-2012 | 441 | 3440 | Population-based |

Supplementary Table 2. Study-specific mean and quartiles of fiber intake (g/day)

| Study | Mean | SD | 25th perc. | 50th perc. | 75th perc. |
| --- | --- | --- | --- | --- | --- |
| Buiatti et al., 1989 [16] | 35.7 | 9.8 | 28.9 | 34.8 | 41.8 |
| Ward et al., 1997 [17] | 10.7 | 5.1 | 7.5 | 10.4 | 13.2 |
| Lopez Carillo et al., 1999 [18] | 27.0 | 12.3 | 18.3 | 25.3 | 33.3 |
| Zaridze et al., 2000 [19] | 39.4 | 16.8 | 28.6 | 38.4 | 46.7 |
| Setiawan et al., 2005 [20] | 5.8 | 4.9 | 3.4 | 4.9 | 7.3 |
| Lunet et al., 2007 [21] | 23.5 | 8.5 | 17.5 | 22.1 | 27.7 |
| Pakseresht et al., 2007 [22] | 17.7 | 7.2 | 13.3 | 16.6 | 21.6 |
| Lucenterforte et al., 2008 [23] | 15.8 | 5.2 | 12.2 | 15.2 | 19.0 |
| Hernandez Ramirez et al., 2009 [24] | 22.4 | 7.9 | 15.8 | 21.4 | 27.3 |
| Santibanez et al., 2012 [25] | 19.0 | 7.8 | 13.1 | 17.2 | 23.8 |
| Castano Vinyals et al., 2015 [26] | 22.7 | 9.2 | 16.7 | 21.5 | 26.9 |

Perc, percentile

Supplementary Table 3. Study-specific odds ratio for a quartile increase in fiber intake, and meta-analysis

| Study | OR | 95% CI |
| --- | --- | --- |
| Buiatti et al., 1989 [16] | 1.05 | 0.86 - 1.28 |
| Ward et al., 1997 [17] | 0.92 | 0.62 - 1.35 |
| Lopez Carillo et al., 1999 [18] | 0.78 | 0.54 - 1.14 |
| Zaridze et al., 2000 [19] | 0.96 | 0.79 - 1.17 |
| Setiawan et al., 2005 [20] | 0.65 | 0.36 - 1.16 |
| Lunet et al., 2007 [21] | 1.10 | 0.94 - 1.29 |
| Pakseresht et al., 2007 [22] | 0.58 | 0.41 - 0.80 |
| Lucenterforte et al., 2008 [23] | 0.64 | 0.46 - 0.89 |
| Hernandez Ramirez et al., 2009 [24] | 1.13 | 0.79 - 1.61 |
| Santibanez et al., 2012 [25] | 0.97 | 0.76 - 1.24 |
| Castano Vinyals et al., 2015 [26] | 0.76 | 0.61 - 0.93 |
| Meta-analysis* | 0.87 | 0.76 - 1.00 |

OR, odds ratio adjusted sex, age, tobacco smoking, fruit and vegetable intake, total energy intake, socioeconomic status

CI, confidence interval

* p-value test for heterogeneity = 0.005

Supplementary Table 4. Odds ratio for a quartile increase in fiber intake, excluding one variable at the time from the regression model

| Variable excluded from the regression model | OR | 95% CI |
| --- | --- | --- |
| Sex | 0.91 | 0.85 - 0.97 |
| Age | 0.92 | 0.86 - 0.98 |
| Tobacco smoking | 0.91 | 0.85 - 0.97 |
| Fruit and vegetable intake | 0.82 | 0.78 - 0.87 |
| Total energy intake | 1.06 | 1.00 - 1.12 |
| Socioeconomic status | 0.92 | 0.86 - 0.98 |

OR, odds ratio adjusted study, sex, age, tobacco smoking, fruit and vegetable intake, total energy intake, socioeconomic status, as appropriate

CI, confidence interval

Supplementary Table 5. Results of model adjusted and unadjusted for additional dietary factors (vegetables and legumes intake, alcohol intake, meat intake).

|  | Vegetables and legumes* | Alcohol | Analysis within the same subset of studies with alcohol variable, without adjusting for alcohol in the model | salt | Analysis within the same subset of studies with salt variable, without adjusting for salt in the model | Meat | Analysis within the same subset of studies with meat variable, without adjusting for meat in the model |
| --- | --- | --- | --- | --- | --- | --- | --- |
| Fiber intake | OR, 95% CI | OR, 95% CI | OR, 95% CI | OR, 95% CI | OR, 95% CI | OR, 95% CI | OR, 95% CI |
| Q1  Q2  Q3  Q4 | Ref  0.79, 0.69-0.92  0.72, 0.61-0.86  0.74, 0.61-0.90 | Ref  0.92, 0.78-1.08  0.83, 0.68-1.01  0.83, 0.67-1.04 | Ref  0.91, 0.77-1.07  0.81, 0.67-0.98  0.80, 0.62-1.00 | Ref  0.78, 0.67-0.91  0.71, 0.59-0.86  0.69, 0.55-0.87 | Ref  0.78, 0.67-0.91  0.72, 0.59-0.87  0.70, 0.56-0.88 | Ref  0.91, 0.77-1.07  0.86, 0.70-1.04  0.88, 0.70-1.11 | Ref  0.78, 0.67-0.90  0.70, 0.59-0.83  0.71, 0.58-0.87 |
| For one quartile of increase in fiber intake | 0.92, 0.86-0.98 | 0.94, 0.88-1.01 | 0.93, 0.87-1.00 | 0.89, 0.83-0.96 | 0.90, 0.83-0.97 | 0.96, 0.89-1.04 | 0.90, 0.85-0.97 |

*Analysis performed on the same subset of studies as the main analysis

Studies with data on alcohol: [19,21,22,24,26,28-31]

Studies with data on salt: [ 22-26, 28-31]

Studies with data on meat: [21,23,26-31]
